# Topical betadine exposure precipitating sterile cerebritis and systemic iodine toxicity

**DOI:** 10.1002/ccr3.7693

**Published:** 2023-07-18

**Authors:** Cody J. Rasner, Timothy Allen, Meredith Kavalier

**Affiliations:** ^1^ University of Minnesota Medical School Minneapolis Minnesota USA

**Keywords:** encephalopathy, iodine toxicity, squamous cell carcinoma, sterile cerebritis

## Abstract

Cerebritis may present with encephalopathy, seizures, localizing neurologic deficits, or death. Topical betadine has a high iodine content and application to open scalp wounds may cause sterile cerebritis.

## Graphical abstract

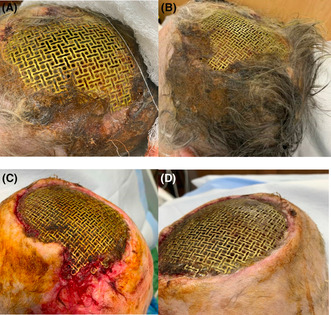

## 1 BACKGROUND

Cerebritis is a potentially fatal infectious or inflammatory condition that presents with symptoms of encephalopathy, seizures, or localizing neurologic deficits.

Betadine (or povidone‐iodine) is a commonly used antiseptic that, once applied, penetrates microbial cell membranes, interacts with proteins, nucleotides, and fatty acids within the cell, and results in rapid cell death.^1^ This mechanism of action promotes a wide antimicrobial spectrum as well as efficacy in disrupting biofilms.^1^, ^2^

Betadine may cause irritant dermatitis when applied to the skin, however, the effect of betadine on neural tissue is less understood. Li et al. describe histologic evidence of rabbit neural tissue damage with increasing concentrations of betadine in the absence of clinical indications of such damage.^3^ Wang et al. also noted that lower concentrations of betadine (eg. 0.5%) were beneficial for tissue healing but higher concentrations (eg. 5% or more) had the opposite effect.^4^ To our knowledge, there are no reports of iodine toxicity resulting in sterile cerebritis secondary to recurrent topical betadine application.

## 2 OBJECTIVE

Here we report an interesting presentation of sterile cerebritis precipitated by chronic topical betadine application during the care of an open scalp wound.

## 3 CASE REPORT

A 73‐year‐old woman with a pertinent past medical history of diabetes and squamous cell carcinoma (SCC) of the scalp presented with her family to her primary care provider for her annual Medicare visit 2 days prior to hospitalization. During the visit, her family reported increased confusion and falls over the course of 2 weeks as well as increased foul smelling, purulent drainage emanating from a surgical site wound on her scalp. Review of systems was negative for fever, chills, headaches, dizziness, hypoglycemia, or other infectious symptoms. She reported taking her medications as prescribed. In addition, her PHQ score was 18 (her score was previously one, 14 months prior to presentation).

Physical exam revealed normal vital signs and an intact cranial surgical mesh with an adjacent area of hypertrophied, hardened skin and purulent drainage. Bloodwork from the visit revealed a mild leukocytosis of 11.9 k/cu mm (4.5–11 k/cu mm), elevated ESR and CRP of 75 and 11.7 (<30 and <0.5, respectively), and an elevated hemoglobin A1c of 10.3% (6.2% 6 months prior to presentation). With these results, a prompt evaluation in the nearest emergency department (ED) was recommended to assess for a central nervous system (CNS) infection.

The SCC of her scalp had previously eroded through her cranium to the dura, resulting in craniectomy with placement of a titanium fenestrated mesh. To close the defect, she underwent two flap procedures (most recently 20 months prior to presentation); however, both attempts were unsuccessful. Not wanting to undergo additional procedures, the defect had been treated for at least 6 months using Betadine 10% solution which was discontinued 2 weeks prior to her presentation.

**FIGURE 1:**
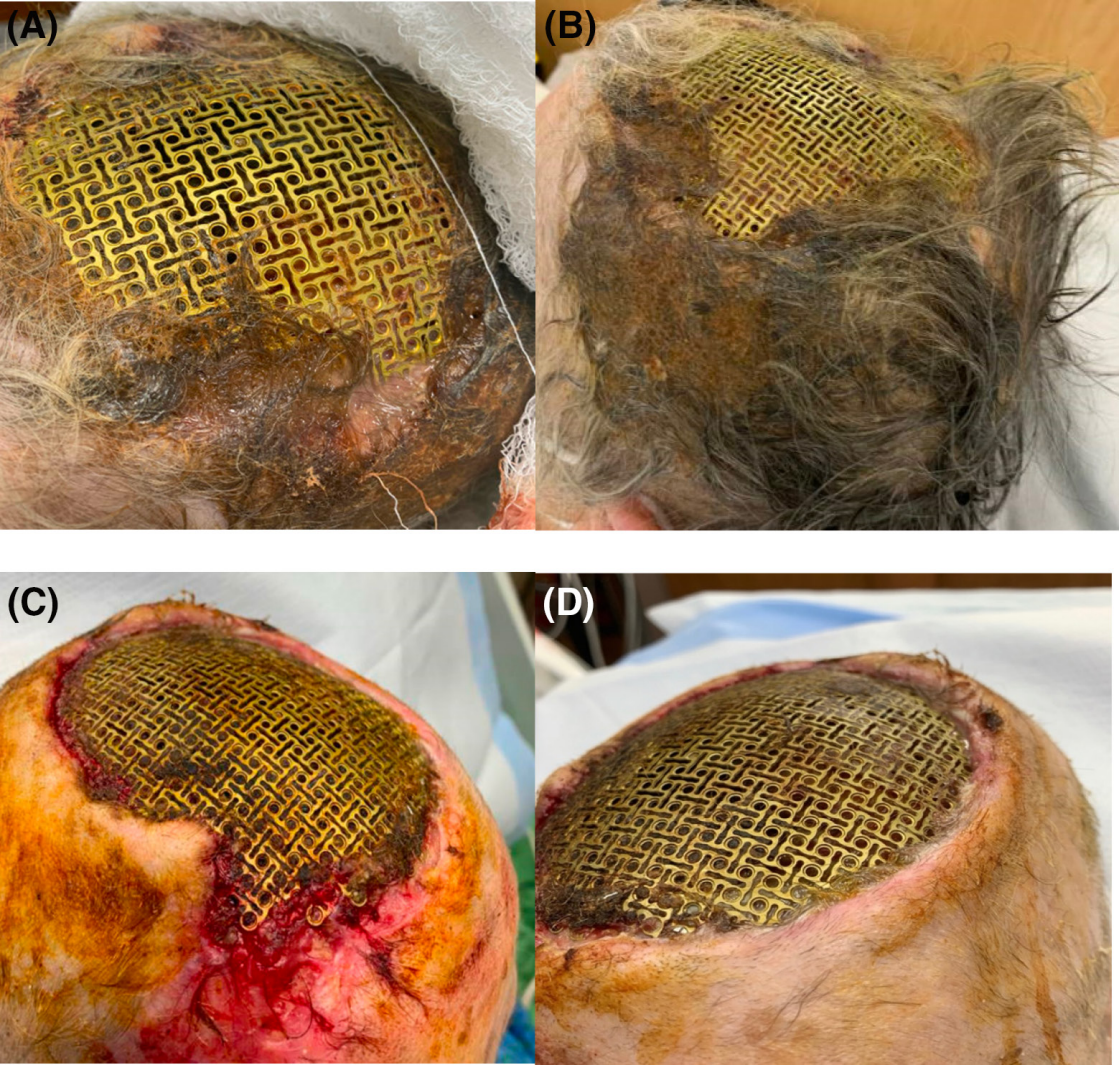
Initial images of patients scalp wounds upon arrival (A and B) and after initial wound cares (C and D).

In the ED, her vitals were: 97.9F, heart rate 69, respiratory rate 20, blood pressure 110/45, and O2 saturation 100% on room air. She was alert and oriented with an intact surgical mesh on her head. The surgical site had clean, dry dressing wrapped around it. The surgical site was surrounded by brown/gray material that appeared to be a mix of matted hair and dried discharge without any obvious purulence, foul smell, or tenderness (Figure 1A,B).

After obtaining initial bloodwork (Table 1) and blood cultures, she underwent imaging, a lumbar puncture (Table 2), wound cleaning (Figure 1C,D), and was started on intravenous antibiotics and supportive cares (vancomycin, cefepime, and metronidazole at CNS penetration dosing).

**TABLE 1:** Baseline serologic results and normal reference values.

White Blood Cells	10.4 (normal: 4–11)	Lactate	0.9 (normal: 0.7–2)	Sodium	134 (normal:136–145)
Absolute Neutrophils	9.3 (normal: 1.6–8.3)	Erythrocyte Sedimentation Rate	85 (normal: 0–30)	BUN	42.9 (normal:8–23)
				Creatinine	1.28 (normal: 0.51–0.95)
Hemoglobin	8.0 (normal: 11.7–15.7)			Glucose	230 (normal: 70–99)
Hematocrit	25.6% (normal: 35%–47%)	C reactive protein	116 (normal: <5)	AST	13 (normal: 10–35)
Platelets	282 (normal: 150–450)			ALT	14 (normal: 10–35)

**TABLE 2:** Cerebral Spinal Fluid (CSF) laboratory results and normal reference values.

CSF Gross Description	Tube number 4 Colorless Clear	CSF Aerobic Bacterial stain and culture	+1 WBC No organisms No growth
Total Nucleated Cells	0	CSF Anaerobic Bacterial culture	No growth
RBC Count	0	CSF Fungal culture	No growth
CSF Glucose	111 (normal: 40–70)	CSF Meningitis panel	Negative
CSF Protein	44.2 (normal: 15–45)	CSF Encephalitis panel	Negative

**FIGURE 2:**
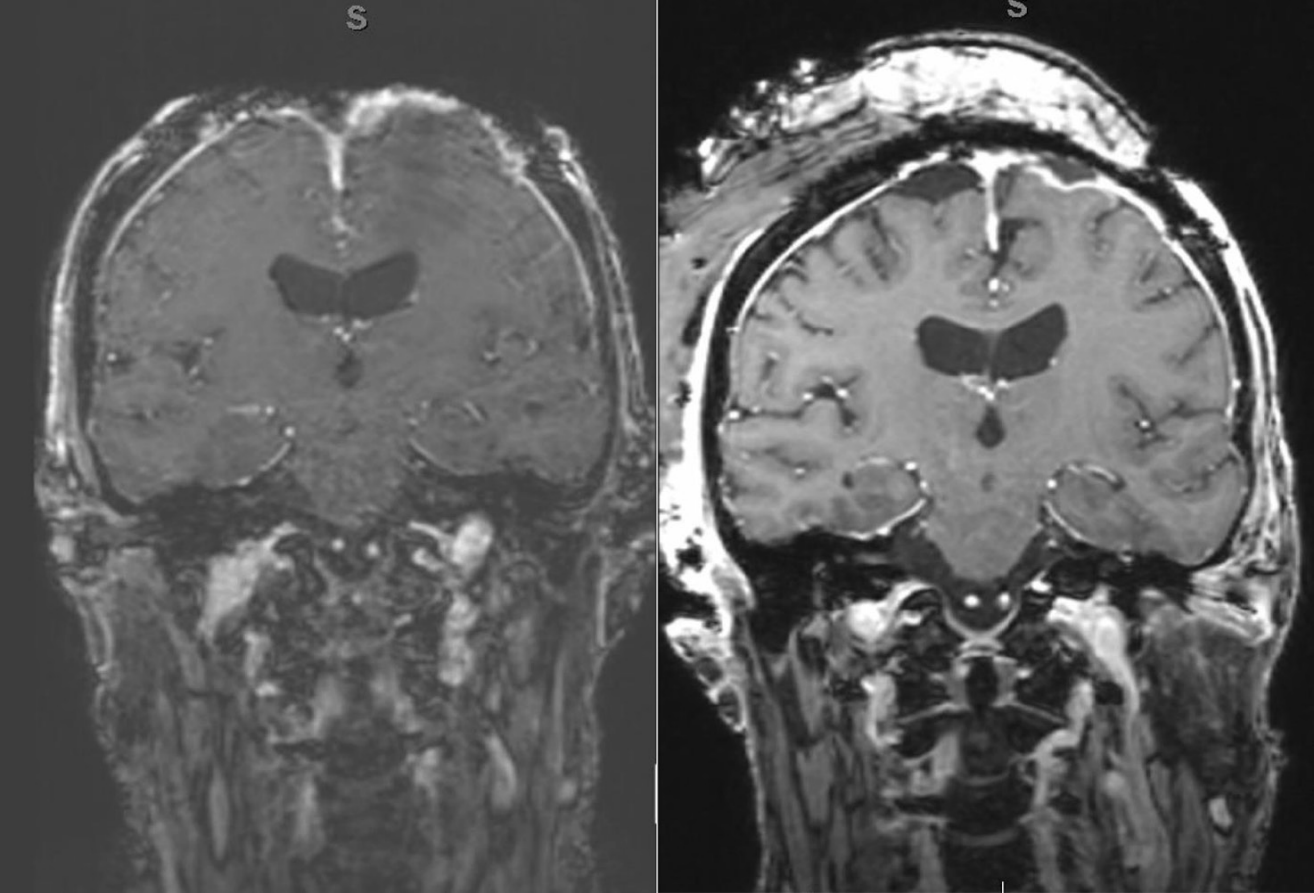
Neuroradiologic magnetic resonance imaging during acute cerebritis (Left) and similar imaging obtained 1 year prior (Right).

Magnetic resonance imaging (MRI) of her brain revealed left frontal cerebritis with associated bifrontal leptomeningitis and pachymeningitis without any intra‐ or extra‐axial abscess. After discussing the case with Neuroradiology and comparing current imaging (Figure 2) to images obtained 1 year prior (Figure 2), there were no significant changes noted in the primary area of concern. A serum iodine level was obtained which returned elevated at 404 nanograms/milliliter (normal: 40–92 ng/mL).

During her hospital course, her creatinine improved with intravenous fluids and was determined to be secondary to dehydration from poorly controlled diabetes. She declined any additional procedures to close the defect. Despite being on broad spectrum antibiotics, she began to experience new episodes of confusion. Repeat imaging revealed new enhancement of the scalp overlying the craniectomy defect as well as extra‐axial abscess formation, concerning for a worsening scalp infection with involvement of the mesh. The patient was ultimately discharged home on hospice with empiric oral antibiotics for the scalp soft tissue infection and new wound care instructions (using Vashe moistened sterile gauze rather than betadine to avoid worsening of condition).

## 4 DISCUSSION

Betadine is a topical antimicrobial agent with a large iodine content frequently used for wound care. Excessive iodine intake has well‐characterized toxicities within the body. Specifically, elevated serum levels of iodine may lead to thyroid dysfunction resulting in both psychological and metabolic effects. In this patient, iodine likely permeated tissues during wound care therein entering the meninges. Furthermore, iodine absorption is enhanced on denuded skin or other tissues without a protective barrier.^5^ Every milliliter of betadine 10% contains 10 mg of iodine. Once iodine is absorbed, it reaches equilibrium rapidly with the extracellular space and distributes evenly except in certain areas, one of which is the thyroid where it is used to make thyroxine (T4) and triiodothyronine (T3). Excessive iodine may result in a transient reduction in thyroid hormone synthesis (Wolff‐Chaikoff effect) by inhibiting thyroid peroxidase (TPO) activity.^6^ Iodine also prevents the release of preformed thyroid hormones (called the Plummer effect) by inhibiting proteolysis of thyroglobulin.^6^ Upon checking her thyroid status, she was found to have a low normal TSH at 0.44 (0.3–4.2) and a low normal FT4 at 1.1 (0.9–1.7).

A reduction in thyroid hormone influences other organs, such as the pancreas and the brain. In the pancreas, it may lead to decreased insulin secretion, via decreased stimulatory signal on pancreatic beta cells, thus leading to hyperglycemia and worsening of diabetes.^7^ In the brain, reduced thyroid hormones may result in pseudo‐depression due to alterations in metabolic activity of neurons.^8^ Thinking back at our patient's initial presentation to her PCP, we suspect that the systemic absorption of iodine from betadine was exacerbating her chronic medical conditions leading to her worsening mood and poor glycemic control despite taking her medications as prescribed. Given that the half‐life of iodine is about 66 days, and she reported stopping it 2 weeks prior to presentation, we theorize that her actual plasma iodine level was much higher.

Given the lack of significant differences seen on imaging as well as her benign physical exam and lab results, we suspected that the patient had a chronic sterile cerebritis due to the Betadine used in wound cares and an acute soft tissue infection of the scalp. We theorized that the irritation seen on imaging resulted from Betadine exposure via seepage thru the cranial mesh fenestration. To confirm this theory, a serum iodine level was obtained which returned elevated at 404 (normal: 40–92). Unfortunately, we were unable to obtain an iodine level from the CSF as no such test exists.

In conclusion, we suspect, and have evidence to support, that direct application of betadine to neural tissue in humans can lead to systemic iodine toxicity (serum level of 404) with down‐stream effects such as worsening glycemic control (HbA1c increased from 6.2% to 10.3% over 6 months) and neurologic dysfunction (presentation with altered mental status, PHQ‐9 score dropping from 1 to 18). While the betadine functioned appropriately in the prevention of infectious cerebritis (as evidenced by the lumbar puncture results and patient's clinical stability), it did have unintended consequences for our patient and should be avoided in similar clinical scenarios.

## AUTHOR CONTRIBUTIONS

**Cody J. Rasner:** Conceptualization; methodology; visualization; writing – original draft; writing – review and editing. **Timothy Allen:** Conceptualization; data curation; investigation; supervision; writing – original draft; writing – review and editing. **Meredith Kavalier:** Conceptualization; data curation; formal analysis; investigation; methodology; project administration; resources; supervision; writing – original draft; writing – review and editing.

## FUNDING INFORMATION

This study received no external funding.

## CONFLICT OF INTEREST STATEMENT

The authors declare no conflicts of interest.

## ETHICS STATEMENT

This study was conducted in accordance with the ethical standards of the University of Minnesota Medical School.

## CONSENT STATEMENT

Written informed consent was obtained from the patient's family for the publication of this case report.

## Data Availability

The data that support the findings of this study are available from the corresponding author upon reasonable request.
